# Nitrates/Nitrites in Food—Risk for Nitrosative Stress and Benefits

**DOI:** 10.3390/antiox9030241

**Published:** 2020-03-16

**Authors:** Małgorzata Karwowska, Anna Kononiuk

**Affiliations:** Department of Meat Technology and Food Quality, University of Life Sciences in Lublin, ul. Skromna 8, 20-704 Lublin, Poland; anna.kononiuk@up.lublin.pl

**Keywords:** nitrites/nitrates, food, health effect, nitrosative stress, processed meat

## Abstract

In the context of impact on human health, nitrite/nitrate and related nitrogen species such as nitric oxide (NO) are a matter of increasing scientific controversy. An increase in the content of reactive nitrogen species may result in nitrosative stress—a deleterious process, which can be an important mediator of damage to cell structures, including lipids, membranes, proteins and DNA. Nitrates and nitrites are widespread in the environment and occur naturally in foods of plant origin as a part of the nitrogen cycle. Additionally, these compounds are used as additives to improve food quality and protect against microbial contamination and chemical changes. Some vegetables such as raw spinach, beets, celery and lettuce are considered to contain high concentrations of nitrates. Due to the high consumption of vegetables, they have been identified as the primary source of nitrates in the human diet. Processed meats are another source of nitrites in our diet because the meat industry uses nitrates/nitrites as additives in the meat curing process. Although the vast majority of consumed nitrates and nitrites come from natural vegetables and fruits rather than food additives, there is currently a great deal of consumer pressure for the production of meat products free of or with reduced quantities of these compounds. This is because, for years, the cancer risks of nitrates/nitrites have been considered, since they potentially convert into the nitrosamines that have carcinogenic effects. This has resulted in the development and rapid expansion of meat products processed with plant-derived nitrates as nitrite alternatives in meat products. On the other hand, recently, these two ions have been discussed as essential nutrients which allow nitric oxide production and thus help cardiovascular health. Thus, this manuscript reviews the main sources of dietary exposure to nitrates and nitrites, metabolism of nitrites/nitrates, and health concerns related to dietary nitrites/nitrates, with particular emphasis on the effect on nitrosative stress, the role of nitrites/nitrates in meat products and alternatives to these additives used in meat products.

## 1. Introduction

Nitrate and nitrite ions are widespread in the environment and occur naturally in plant foods (vegetables) and water. The contribution of drinking-water to nitrate intake is usually low (less than 14%). However, due to the use of inorganic fertilizer, nitrate levels in water resources have increased in many places of the world, recently. In this context, in a situation where nitrate concentrations in drinking-water are below 10 mg/L, food (mainly vegetables) will be the main source of nitrate for the human. In the reverse situation, when the nitrate level in drinking-water is high (exceeding 50 mg L^−1^), water will definitely be the main source of exposure to nitrates [1,2].

Nitrates/nitrites can also be used as additives in food of animal origin. Nitrites (sodium nitrite—E249, potassium nitrite—E250) and nitrates (sodium nitrate—E251, potassium nitrate—E252) are authorized as food additives in the European Union under Commission Regulation (EU) No 1129/2011. They are used in food to stabilize processed meat and cheese. The regulation determines the maximum amount of nitrites and nitrates that may be added as a food additive during food processing. The amount of nitrite permitted for use in processed meat is currently 150 mg kg^−1^, with the exception of sterilized meat products for which the limit is 100 mg kg^−1^. The addition of sodium nitrate is allowed only in uncooked meat, to a maximum amount of 150 mg kg^−1^. Nitrites can also be present in dairy products from exogenous sources. The maximum concentration of nitrite allowed in the regulation for cheese is 150 mg kg^−1^.

Due to these sources of nitrates/nitrites, humans are exposed to these compounds. Some studies have estimated exposure to nitrites and nitrates [3,4,5]. According to the European Commission’s former Scientific Committee for Food (SCF) and the Joint FAO/WHO Expert Committee on Food Additives (JECFA), the current acceptable daily intake (ADI) for nitrites are 0.06 and 0.07 milligrams per kilogram of body weight per day, respectively. In the case of nitrates, both organizations establish the ADI at 3.7 mg/kg bw/day.

Nitrate intake with food is associated with some health risks. When these compounds are consumed, about 60%–70% is easily absorbed and rapidly excreted in urine. In humans, about 3% of nitrate appears in urine as urea and ammonia. Nitrates may also survive passage through the stomach and enter the circulatory system. A variety of highly bioactive reactive nitrogen oxide species are formed under acidic gastric conditions or in blood and tissues. These may be involved in the generation of nitrosamines of toxicological importance when there are secondary amines present in the stomach [6]. According to Ding et al. [6], the presence of dietary antioxidants inhibits the generation of nitrosamines. The process of nitrosamine formation was completely inhibited when the molar ratio of antioxidants and nitrite was higher than 2:1.

This manuscript on nitrates and nitrites in food will review the main sources of dietary exposure to nitrates and nitrites, metabolism of nitrites/nitrates, health concerns related to dietary nitrites/nitrates, the role of nitrites/nitrates in meat products and alternatives to these additives used in meat products.

A systematic and comprehensive article retrieval strategy that provided a general impression of the risk for nitrosative stress and benefits due to nitrate or nitrite consumption was conducted. The Web of Science was searched for articles of studies assessing the relationship between the risk of cancer the nitrate or nitrite consumption. Many relevant articles were obtained by combing the keywords (nitrate, nitrite, risk of nitrosative stress, cancer) in a more detailed retrieval strategy. Moreover, a manual search of the references of relevant articles has been done.

## 2. The Main Sources of Dietary Exposure to Nitrates and Nitrites

**Figure 1:**
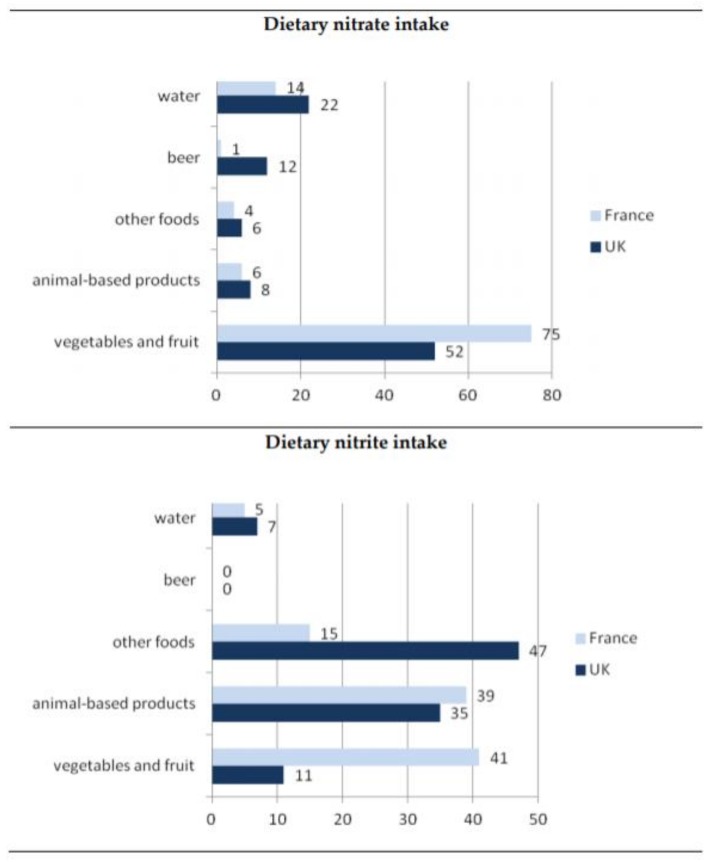
General dietary exposure of nitrate and nitrite [8].

The largest amount of nitrates is accumulated in plants growing in a nitrate-rich environment [7]. Based on available data (Figure 1), the most important sources of dietary intake of nitrate are vegetables and fruit, contributing 50% to 75% to the overall dietary intake for both the UK and France [8]. Several factors affect accumulation of nitrates in plants. Generally, factors such as applications of fertilizers, nitrate reductase activity, growth rate and growth conditions, including intensity of light, level of rainfall, significantly affect the nitrate content in vegetables [9]. Research shows that leafy vegetables tend to have higher levels of nitrates compared to seeds or tubers. Therefore, rucola, lettuce and spinach have the highest nitrate content (Table 1). Beets and celery are also examples of vegetables containing a significant amount of nitrates. Moreover, as indicated by Lucarini et al. [10] the content of nitrates in vegetables is strongly influenced by seasonality and by the cultivation systems. Their findings indicated that lettuce biodynamically grown accumulated 1.3–2 times less nitrate than the respective organically grown plants.

Maximum levels for nitrate in vegetables, set in the EU has been amended several times. The current maximum levels are laid down in Regulation (EC) N. 1258/2011. The Regulation applies for the following foodstuff: spinach, lettuce, rocket and processed cereal-based foods and baby foods for infants and young children. All maximum levels are expressed as mg nitrate kg^−1^ fresh weight.

Studies have assessed that processing methods such as heat treatments and storage conditions cause the loss of nitrates. The effect of increased storage temperature on decreasing the nitrite content of vegetables has been indicated due to increased bacterial facilitated reduction of nitrates to nitrites [11]. Research carried out by Ding et al. [6] on the evaluation of nitrate and nitrite contents in pickled fruit and vegetable products indicated that nitrate content in pickled products (among others pickled beets, cauliflowers, carrots, Brussels sprouts) was generally lower compared to fresh fruits and vegetables. A reduction in nitrates was expected by these authors due to the production processes used (acidification, brining, pasteurization and shelf-stable).

According to WHO [12], humans generally consume between 1.2 and 3.0 mg of nitrite daily. Based on Nuñez de González et al. [13] research, approximately 80%–85% of human exposure to nitrates comes from vegetables. Other sources of nitrates in the human diet are fruits, cereals, water, meat products, and therapeutic treatments for angina and digital ischemia [13]. Larsson et al. [3] examined the intake of nitrates and nitrites in Swedish children. Based on their results, the mean intake of nitrites from cured meat among children in the age of 4–12 years was 0.007–0.13 mg kg^−1^ body weight per day while the mean intake of nitrates from several sources together including vegetables, fruit, cured meat and water was from 0.45 to 0.84 mg kg^−1^ body weight per day for the same groups of children. So, given the food sources, no child exceeded the ADI. However, when the total nitrite intake was included (an estimated 5% endogenous conversion of nitrates to nitrites) approximately 12% of the four-year-old children exceeded the nitrite ADI.

Temme et al. [4] assessed the dietary intake of nitrates and nitrites in Belgium. Processed vegetables, cheeses and meat products were taken into consideration as a source of nitrites/nitrates. In case of Belgian population, the mean usual daily intake of nitrates was 1.38 mg kg^−1^ bodyweight per day what means that exposure to nitrates at a mean intake constituted 38% of the ADI. The authors showed that half of the intake was from vegetables (especially lettuce), followed by from water and water-based drinks (20%). The average daily intake of nitrates and nitrites whose sources were cheese and meat products was 0.2% and 6% of the ADI, respectively. Research on nitrate/nitrite intakes by the Estonian population conducted by Tamme et al. [5] showed the highest mean values of nitrates in dill (2936 mg kg^−1^), spinach (2508 mg kg^−1^), lettuce (2167 mg kg^−1^) and beetroot (1446 mg kg^−1^). The mean intake of nitrates by the Estonian population was 58 mg day^−1^, while the average daily intake of nitrates by children (4–6 years) was 30 mg. The authors also estimated infants’ average daily intake of nitrates from consumption of foods based on vegetables—this was 7.8 mg.

Thus, the fact that the consumption of vegetables is increasing due to dietary recommendations requires reflection. The World Cancer Research Fund/American Institute for Cancer Research rates the evidence on diets high in vegetables and/or fruits in the context of protection against a variety of cancers. This research considered whether this effect is also related to high nitrite content [16].

## 3. Health Concerns Related to Dietary Nitrites/Nitrates

### 3.1. Metabolism of Nitrites/Nitrates

The major sources of nitrates and nitrites for humans are diet and endogenous formation. Nitrates and nitrites in the human organism are partially excreted as well as circulating and reduced to nitrite and nitrogen oxides which ensures a nitrate-nitrite-NO balance [17,18].

**Figure 2:**
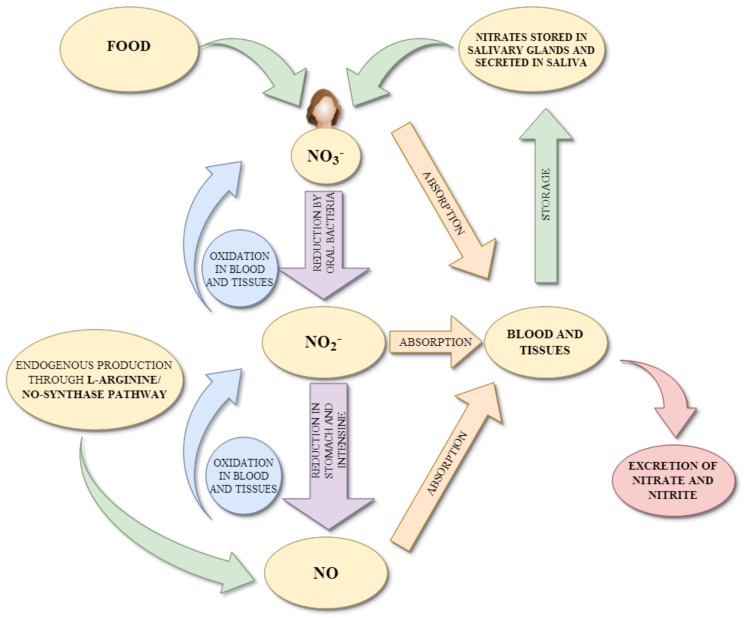
Simplified scheme of nitrite and nitric oxide metabolism in the body.

Endogenous nitrates and nitrites are produced in the L-arginine/NO-synthase pathway [19]. They are an end product of NO oxidation. NO is formed in endothelial cells, where L- arginine is metabolized to citrulline with the formation of NO in the pathway catalyzed by nitric oxide synthases (NOSs) [20]. Released NO is a highly reactive compound, so an excess of NO is rapidly oxidized (in blood) to nitrites and nitrates by oxyheme proteins (oxyhemoglobin or oxymyoglobin) [21]. The production of NO through the L-arginine/NO-synthase pathway is inhibited under hypoxia and ischemia conditions, then dietary nitrates and nitrites are the only effective donors of NO [22]. Dietary and saliva nitrates are partially reduced into nitrites and subsequent to biologically active nitrogen oxides (to perform physiological functions) in the nitrate-nitrite-NO pathway. Approximately 5%–7% of dietary nitrates and 20% of salivary nitrates are reduced to nitrites in the oral cavity by commensal bacteria [22,23,24,25]. Oral bacterial species, located at the back of the tongue, catalyzed by nitrate reductase enzymes (NOSs), reduce nitrates to nitrites. After swallowing, nitrates and nitrites in the gastric acidic milieu in the stomach are metabolized in various enzymatic (deoxyhemoglobin, myoglobin, neuroglobin, xanthine oxidoreductase, aldehyde oxidase, carbonic oxidase, and mitochondrial enzymes) and non-enzymatic (pH-dependent reduction) systems to NO as well as other biologically active nitrogen oxides (such as N_2_O_3_, NO_2_) [26,27]. Additionally, dietary compounds such as vitamin C and polyphenols can enhance the formation of NO from nitrites as well as protect NO against oxidative destruction (prolong the half-life of NO) [28,29]. Next, in the small intestine they are systemically absorbed and end up in blood and plasma. NO present in blood and tissues can be further spontaneously oxidized forming nitrites and nitrates. Excess nitrates are excreted by urine (approximately about 75% of total nitrate), whereas the rest is reabsorbed and concentrated in salivary glands and then secreted in saliva [30]. The concentration of nitrates in salivary glands is 10–20 fold higher than in plasma [23]. Figure 2 presented the scheme of nitrite and nitric oxide metabolism in the body.

Nitrates are unstable in acidic conditions, and so spontaneously decompose to nitrites and nitrogen dioxide. Thus, nitrites resulting from nitrate metabolism as well as those supplied with food can additionally react in the gastrointestinal tract with precursors of N-nitroso compounds (such as amines and amides) and result in the formation of N-nitroso compounds [31]. Reaction of nitrites with secondary amines is considered especially dangerous because it leads to the formation of carcinogenic nitrosamines. Primary amines with nitrites only form unstable nitrosamines, which are immediately degraded to alcohol and nitrogen, whereas tertiary amines do not react with nitrites [23,32]. It is estimated that endogenous nitrosation leads to the formation of 45%–75% of total human exposure to N-nitroso compounds [33]. Additionally, low pH in the stomach and the presence of iron (gastric juice contains a significant amount of iron) are factors that significantly enhance the nitrosation process [34]. Other documented factors that influence endogenous N-nitroso compound formation are vitamin C and E (considered inhibitors of this process) [35] and heme iron (considered a stimulant) [36].

### 3.2. Effect on Nitrosative Stress

**Figure 3:**
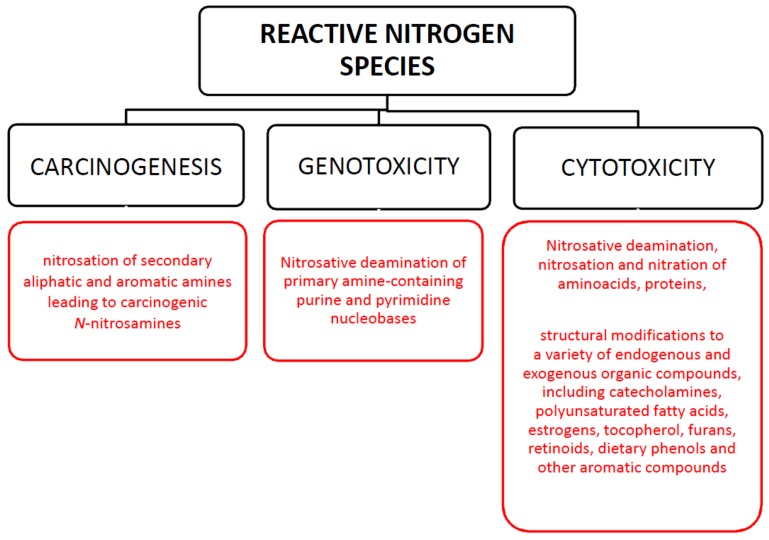
Toxicological effects of reactive nitrogen species.

Nitrite plays a distinct role in human physiology. Some of the physiological properties associated with NO derived from nitrite in humans are connected with arterial blood pressure, immune response, biofilm formation. However, in acidic environments or under oxidative stress conditions it can be converted to a range of reactive nitrogen species (RNS) [37]. Oxidative/nitrosative stress as a result of the increase in the content of reactive oxygen/nitrogen forms is recognized in turn as a prominent feature of many acute and chronic diseases [38]. Nitrosative stress levels are mainly related to the concentration and exposure time to RNS as well as the ability of cellular antioxidants to remove them [39]. The reactive nitrogen species include nitric oxide (NO), nitrogen dioxide (NO_2_), and peroxynitrite (ONOO^−^). NO_2_ and ONOO^−^ are the most highly reactive species [40]. In living systems, their formation is regulated due to the fact that they are involved in a variety of biological functions. However, its unchecked intracellular presence produces significant toxicity as it can target a variety of biomolecules including proteins, DNA, lipids, and carbohydrates [41]. Thus, nitrite and NO occurrence at relatively high levels under nitrosative stress conditions may be associated with a series of adverse events, such as mutagenesis, carcinogenesis (Figure 3).

Nitric oxide is very unstable in an aqueous environment. In the extracellular environment, NO reacts with water and oxygen to form nitrite and nitrate anions. An important path of nitric oxide degradation is the reaction with superoxide anion to form peroxynitrite (ONOO^−^) that is more reactive. Peroxynitrite can form nitrotyrosine, characteristic marker of nitrosative stress, by reacting with proteins. Increased levels of nitric oxide and nitrotyrosine are associated with variety of human skin diseases (skin cancers, systemic lupus erythematosus, psoriasis, urticarial, atopic dermatitis) [42]. Some researchers [43,44] point to the importance of polyunsaturated fatty acids (PUFAs) as potential targets of reactive nitrogen species in vivo as nitrated PUFAs have been identified in human physiological fluids. Nitro-fatty acids are present in the vasculature at nanomolar to low micromolar concentrations. Such concentration is sufficient to exert biological effects such as inhibition of platelet and macrophage activation, proinflammatory cytokine secretion and proliferation of vascular smooth muscle cell [45]. Nitrosative stress-induced peroxidation of membrane lipids can be particularly harmful because it changes the degree of membrane fluidity and increase tissue permeability due to inactivation of membrane-bound receptors or enzymes. Moreover, lipid peroxidation process generates variety of relatively stable products, which can be measured in plasma and urine as an indirect index of nitrosative stress, mainly α, β-unsaturated aldehydes (malondialdehyde, 4-hydroksy-2-nonenal, 2-propenal). Some of these products have been proven to exhibit facile reactivity with proteins, DNA and phospholipids leading to the generation of products that contribute to the pathogenesis of many diseases. The clinical relevance of the reaction between malondialdehyde and proteins is highlighted in atherosclerosis while 4-hydroksy-2-nonenal exhibits numerous cytotoxic, mutagenic, genotoxic effects including inactivation of enzymes, inhibition of proteins and DNA synthesis [46]. As stated by White et al. [44], lipid reactions with reactive nitrogen species can cause formation of *cis* or *trans* nitro-alkanes, where the NO_2_ group is present at the site of the double bond, as well as nitro-hydroxy and nitro-hydroperoxy lipids.

Proteins are also major targets for reactive nitrogen species. Exposure of proteins to RNS cause major physical changes in protein structure and thus have a wide range of functional consequences including inhibition of enzymatic and binding activities, increased susceptibility to aggregation and proteolysis and altered immunogenicity [38]. As reported by Dalle-Donne et al. [38] increased concentration of nitrated plasma proteins has been linked with unfavorable outcome on development of lung injury. Patients with lung cancer have significantly higher serum concentration of nitrated proteins confirming the presence of nitrosative stress. As concluded by White et al. [44], recently published results showed that tyrosine nitration and S-nitrosylation of insulin-signaling intermediates create novel means to modulate metabolic functions in insulin target cells.

**Figure 4:**
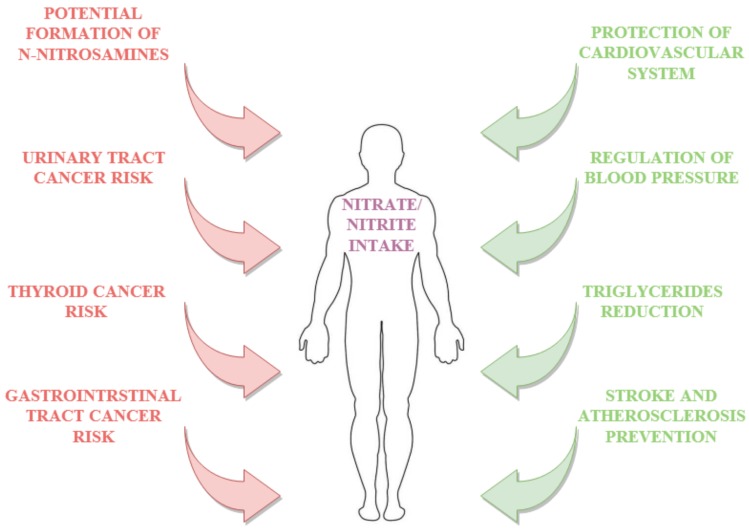
Selected benefits and adverse effects on nitrate nitrite intake.

The effect of dietary nitrates and nitrites is associated with cancer risk (Figure 4). Nevertheless, published results of human studies on the relationship between nitrate and nitrite intake and cancer risk are inconsistent. On the one hand, there is a lot of evidence of a connection between nitrate and nitrite intake and a higher relative risk (RR) (above 1) of breast cancer [46], gastric cancer [47,48,49,50,51], renal cell carcinoma [50,51,52], adult glioma [53], colorectal cancer [51,54,55], esophageal cancer [49,55] and thyroid cancer [48]. On the other hand, a recent meta-analysis of epidemiological studies indicated a weak association between dietary nitrate and cancer risk, whilst in the case of dietary nitrites, the dependence was more noticeable [51,56,57]. The authors emphasize the difficulty in risk assessment due to the multiplicity of interactions between dietary components as well as population diversity. The authors also indicated the significance of the source from which the nitrates and nitrites originate. For example, Song et al. [57] conducted an impressive meta-analysis of the literature on the impact of dietary nitrate, nitrite and nitrosamine intake on gastric cancer risk. According to their review, the association between the dietary nitrate, nitrite and nitrosamine intake and the risk of stomach cancer (measured as relative risk—RR) varied between 0.69–0.93, 1.13–1.52 and 1.02–1.76, respectively. Furthermore, intake of nitrates under 600 mg per day is associated with a relative risk under 1, whereas in the case of nitrites and nitrosamines daily intake above 0.2 mg and 0.2 μg is associated with a relative risk of gastric cancer above 1. Their detailed analysis of published results suggests an association between dietary nitrate intake and reduction of gastric cancer, whereas consumption of nitrites and N-nitrosamines increases that risk [57]. They justified this phenomenon by the fact that dietary nitrates were mainly provided by vegetables, and any protective effect may reflect other protective compounds (such as antioxidants or vitamins) and not nitrates. Results obtained by DellaValle et al. [51], Grieb et al. [52] and Hu et al. [58] indicate that dietary intake of nitrates and nitrites from animal sources (including also processed meat) is positively associated with renal cell carcinoma (RCC), whereas in the case of overall nitrate and nitrite intake, including from plant sources, they did not find any associations). Tests carried out by Ward et al. [48] indicate an increased risk of thyroid cancer in people consuming higher amounts of nitrites. NO in high levels may cause chromosomal breaks directly, or by inhibiting DNA repair activities. Nitric oxide can cause irreparable damage to several basic cancer control genes. Superoxide and substance react immediately to form peroxynitrite, and thus influence oxidative DNA damage. NO can also block DNA synthesis inhibiting the rate-limiting enzyme in DNA production (ribonucleotide reductase).

Daily nitrate intake not exceeding 17.4 mg was assessed at a relative risk of 1.00, between 17.5–27.7 mg/day nitrate as RR of 1.65, between 27.8–41.1 mg/ day nitrate as 1.69, whereas daily intake above 41.1 mg/day nitrate is equal to a relative risk at a level of 2.85. Similarly, Aschebrook-Kilfoy et al. [59] described the connection between animal origin nitrite intake and thyroid cancer risk, explaining this by the specificity of meat as a source of precursors for specific N-nitroso compounds which can cause thyroid cancer. It is possible that animal sources of nitrates/nitrites, which are rich in amines and amides as well as heme iron could be the reason for increased production of endogenous N-nitroso compounds, which may contribute to an increase in cancer risk [51,60,61,62]. Due to the formation of nitrosamine compounds, a large amount of which are considered to be carcinogenic, cancer risk is the most serious adverse effect of nitrate and nitrite intake [33]. Nitrates and nitrites are not themselves carcinogenic; nevertheless, they have the potential (during the endogenous pathway as well as processing of food) to react with other compounds to form carcinogens.

Increased dietary exposure to nitrates and nitrites, due to the formation of nitrosamines, can lead to toxicity in the form of methemoglobinemia. When nitrites react with hemoglobin, rendering it incapable of carrying oxygen, biochemical anemia occurs and causes cyanosis. Infants are particularly vulnerable to methemoglobinemia, due to the high intake of nitrate-rich vegetables as well as the fact that only after three-months of age does the body start to produce an enzyme that restores the oxygen-carrying ability of hemoglobin [63]. Nevertheless, according to exposure studies, the hypothesis that nitrates and nitrites are associated with hemoglobinemia is debatable due to the fact that there is no scientific evidence to support it [63].

### 3.3. Benefits

Benefits from dietary nitrate and nitrite intake have been demonstrated in many studies (Table 2). The positive effect of nitrates and nitrites is related to the fact that they are exogenous donors for NO formation, which have a potentially beneficial role in physiology and therapeutics [64]. The most widely considered and described advantage of nitrate and nitrite intake is its positive effect on the cardiovascular system.

The impact of nitrate and nitrite intake on endothelial function and blood pressure is widely studied [65,66,67]. Tests carried out on animals provide evidence that dietary nitrates and nitrites decrease blood pressure through their antioxidant properties. This reduction of blood pressure by nitrates and nitrites is dependent on the conversion of nitrates to nitrites and to NO [68,69,70,71,72,73,74,75,76,77]. The systematic review and meta-analysis published by Siervo et al. [71] showed that clinical studies also confirmed the positive effect of dietary nitrate and nitrite intake. Nevertheless, because the main source of nitrates is plants, studies into hypertension prevention have mainly focused on nitrates and nitrites from this source. Results presented by Ashworth et al. [67] indicated that consumption of two portions of high nitrate vegetables daily for seven days resulted in a reduction of systolic blood pressure of approximately 4mmHg in normotensive women. Additionally, the authors suggested that, in the case of people with hypertension, a greater reduction in blood pressure could be achieved. Kapil et al. [65] examined the impact of daily intake of nitrates (from beetroot juice) on blood pressure and vascular function. The authors also indicated a reduction in blood pressure (250 mL of juice daily, 4 weeks treatment) of 7.7 mm Hg and 5.2 mm Hg for systolic and diastolic blood pressure, respectively, as well as a reduction in pulse wave velocity and augmentation index [66]. Dietary nitrates and nitrites can potentially improve glucose and insulin tolerance. Studies on type 2 diabetic rats clearly indicated that after 2–8 months of taking water containing 100 mg L^−1^ of nitrate, glucose and insulin tolerance, insulin resistance and sensitivity as well as lipid profiles improved, whereas fasting glucose and insulin decreased [72,73]. Nevertheless, the results of clinical studies do not confirm this phenomenon. Supplementation for 14 days with 250 mL day^−1^ beetroot juice does not influence insulin resistance and glucose disposal in type 2 diabetic patients [74,75]. However, results obtained by Zand et al. [76] in clinical studies confirmed a reduction of triglycerides (TG) in patients with TG levels greater than 150 mg/dL, after 30 days of supplementation with high nitrate products. Findings from other clinical studies also suggest that dietary nitrates improve endothelial dysfunction and vascular stiffness in the elderly with moderately increased cardiovascular risk [77]. A higher nitrate consumption may play a role in stroke and atherosclerosis prevention via the reduction of platelet aggregation [78,79]. Intake of nitrates and nitrites can also effectively protect against ischemia-reperfusion injury due to the anti-oxidative as well as anti-inflammatory properties of nitrates [46,80]. Coggan et al. [81] demonstrated that dietary nitrates can increase the maximal speed and power of human muscle during exercise due to increasing NO availability [82]. In addition, in patients with diseases related to tissues (heart failure, chronic obstructive pulmonary disease), nitrate supplementation significantly enhanced NO bioavailability and extended the time-to-exhaustion as well as reduced exercise diastolic blood pressure [66,81].

## 4. The Use of Nitrites in Meat Processing

### 4.1. Role of Nitrites/Nitrates in Meat Products

The meat industry uses nitrates/nitrites as additives in the meat curing process during which the formation of nitric oxide from nitrites is a prerequisite step for reactions. The decrease in the amount of nitric oxide is due to its reactions with myoglobin and other substrates in meat, including amino acids such as cysteine [85]. About 10%–20% of originally added nitrites, referred to as residual nitrites, is typically present in meat products after production, and this amount of residual nitrites slowly declines during the storage period of cured meat products [15]. The average level of residual nitrites in meat products observed is: in France (50 mg kg^−1^) [86]; USA (4.7 mg kg^−1^) [13]; Denmark (6 mg kg^−1^); Belgium (4 mg kg^−1^) [87] and Iran (13.9 mg kg^−1^) [88].

In 2015, the International Agency for Research on Cancer (IARC) declared processed meat subjected to, inter alia, curing to be a Group 1 carcinogen based on data related to colorectal and stomach cancer. In this context, recent research has focused on finding alternatives for nitrates/nitrites in meat processing.

However, the multifunctional nature of nitrate means that no substance has been found that would fully replace the functions of nitrites or nitrates. The beneficial effects of nitrites/nitrates in cured meat products is related to the positive effect of color enhancement, the development of flavor typical of cured meat, the antimicrobial role and antioxidative effect [89]. Nitric oxide reaction with myoglobin (deoxymyoglobin and metmyoglobin) forms nitrosylmyoglobin complex, which outline the unique cured meat color. The role of nitrite/nitrate in the development of unique cured meat flavor is not fully understood. According to Jira [90], several compounds are formed when nitrite is bound to lipids and proteins. For example, when nitrites are bound to sulfur-containing amino acids of meat proteins, SH-residues with a specific aroma and flavor are formed and contribute to the unique flavor of cured meat. Antimicrobial effect of nitrite is related to inhibiting metabolic enzymes of bacteria, limiting oxygen uptake, and breaking the proton gradient. Nitrite is also well known to suppress the outgrowth of spores of *Clostriduium botulinum* [89]. Moreover, nitrite and nitrate act against lipid oxidation through the oxygen deletion [85]. Nitric oxide can react with radicals (hydroxyl radical, alkoxy radicals, and peroxyl radicals) interrupting radical chain reactions and bind to transitional metals.

### 4.2. Alternatives to Nitrites/Nitrates in Meat Products

Many studies have stated that vegetables and drinking-water contribute in larger amounts to nitrate and nitrite levels in the diet than cured meat [4,84]; however, the meat industry is particularly focused on reducing sodium nitrate levels. The reduction of food additives, especially nitrates, as expected by consumers is one of the most important difficulties faced in the meat industry. Consumers prefer natural additives instead of chemicals in meat product formulations due to health risks related to nitroso compounds. Therefore, studies on the reduction or elimination nitrites or nitrates and the use of natural compounds as nitrite/nitrate alternatives have increased in recent years. Thus, manufacturers of some processed meats have begun to use ‘natural’ sources of nitrates, such as celery juice, or beetroot or spinach extract. However, the nitrates present in vegetables are reduced to nitrites with bacterial cultures and as a result contribute to nitrosamine formation [84]. The reduction of nitrates to nitrites is carried out mainly by bacteria possessing nitrate reductase activity (staphylococci and micrococci), which are naturally present in meat or added during processing [91]. In the process of natural curing with natural sources of nitrate (natural juices, dried fruit and vegetable concentrates) starter culture with nitrate reductase activity to subsequently produce nitrite, e.g., *Staphylococcus carnosus*, are also used.

Several studies have shown that it is both feasible and possibly beneficial to use natural alternatives to nitrates and nitrites in meat products (Table 3). Sindelar et al. [92] conducted research using vegetable juice powder and starter culture as a nitrite replacer in cooked frankfurter-style sausage. They concluded that meat products manufactured with vegetable juice powder and a starter culture (containing *Staphylococcus carnosus*) addition were characterized by the quality and sensory attributes similar to traditionally cured products. Similarly, research performed by Kononiuk and Karwowska [93,94] showed a positive effect of the nitrite substitute used on the quality characteristics of the meat product. They indicated that the addition of acid whey had a similar effect on the tested parameters as nitrate/nitrite in fermented sausages.

## 5. Conclusions

Nitrates and nitrites have recently become two of the most controversial substances present in food, both naturally occurring and derived from the additives used during processing. The majority of nitrates are consumed through vegetables. Nitrate levels in vegetables vary greatly, although leafy vegetables (especially rucola and spinach) contain the highest level of nitrates. Nitrate ion is not toxic, but due to the action of anaerobic bacteria (in gastrointestinal tract) 5%–20% of ingested nitrate is converted to nitrite, which is more toxic. The conversion to nitrite and further metabolism of nitrogen compounds to nitrosamines is related to negative effects of nitrate to consumers since is associated with the risk of gastrointestinal cancer. On the other hand, many reports point to benefits with nitric oxide formed as a result of nitrate conversion, including the control of blood pressure, improving cardiovascular health. The meat industry is particularly associated with the use of nitrates and nitrites as these substances are considered as a multifunctional food-additives in meat curing. Due to potential carcinogenic effect, nitrates and nitrites should be limited in the meat industry. However, finding the perfect alternative to nitrates/nitrites in meat processing is very difficult due to its multifunctional nature.

## Figures and Tables

**Table 1:** Nitrate content in vegetables. EFSA: European Food Safety Authority.

Source	Total Nitrate Content (mg kg^−1^)	References
Spinach	1066	EFSA [8]
	2036	Roila et al. [14]
	2333	Sidelar and Milkowski [15]
Rucola	4677	EFSA [8]
Radish	1297	EFSA [8]
Celery	1103	EFSA [8]
	1544	Sidelar and Milkowski [15]
	1495	Nuñez de González et al. [13]
Rhubarb	2943	EFSA [8]
Lettuce	1324	EFSA [8]
	1079	Roila et al. [14]
	786	Sidelar and Milkowski [15]
Chard	1690	EFSA [8]
	1728	Roila et al. [14]
Beets	2756	Sidelar and Milkowski [15]
	1446	Tamme et al. [5]
Beetroot	1379	EFSA [8]
Carrot	238 296	Roila et al. [14] EFSA [8]
Potato	168	EFSA [8]

**Table 2:** Adverse effects and benefits of dietary nitrites/nitrates.

Adverse Effect	Reference	Benefits	Reference
Gastric cancer	Kim et al. [47] Ward et al. [48] Keszei et al. [49] Xie et al. [83] Song et al. [57] Zheng et al. [62]	Adult glioma	Dubrow et al. [53]; Xie et al. [83]
Colorectal cancer	De Roos et al. [54]; Cross et al. [55]; DellaValle [51]; Cantwell et al. [84] Xie et al. [83]; Van Hecke et al. [60] Bestide et al. [61]	Reduction of blood pressure	Kapil et al. [65]; Berry et al. [66]; Ashworth et al. [67]; Amaral et al. [69]; Ling et al. [70]; Sievro et al. [71]
Esophageal cancer	Cross et al. [55]; Keszei et al. [49]; Xie et al. [83]	Atherosclerosis prevention	Bondonno et al. [78]; Burnley-Hall et al. [79]
Thyroid cancer	Ward et al. [48]; Aschebrook-Kilfoy et al. [59]; Xie et al. [83]; Bahadoran et al. [56]	Protection against ischemia- reperfusion	Yang et al. [46]; Lefer et al. [80]
Renal cell carcinoma	Weyer et al. [50]; DellaVale et al. [51]; Grieb [52]; Xie et al. [83]; Hu et al. [58]	Exercise capacity	Coggan et al. [81] Coggan et al. [82]
Methemoglobinemia	Chan et al. [63]	Stroke prevention	Bondonno et al. [78]; Burnley-Hall et al. [79]
Hypothyroidism	Ward et al. [48]; Xie et al. [83]	Insulin resistance, glucose tolerance	Ghasemi, Ghebi [72]; Khalifi et al. [73]; Gilchrist et al. [74]; Cermak et al. [75]
Breast cancer	Yang et al. [46]; Xie et al. [83]	Reduction of triglycerides	Zand et al. [76]
Nitrosative stress	D’Ischia et al. [37]		

**Table 3:** Alternatives to nitrites/nitrates used in meat products.

Additives	Effects	Type of Product	Reference
beetroot powder	control lipid oxidation and residual nitrite contents	fermented beef sausage	Sucu andTurp [95]
rosemary essential oil /lyophilized extract	lipid oxidation inhibition higher antioxidant activity	pork sausages	Bianchin et al. [96]
celery juice powder + starter cultures	control lipid oxidation color development	ready-to-eat ham	Sindelar et al. [92]
freeze-dried cranberry	control lipid oxidation	fallow-deer fermented sausage	Karwowska, Dolatowski [97]
fermented spinach powder	control lipid oxidation	cured pork loins	Kim et al. [98]
acid whey (liquid and freeze-dried)	proteolysis changes food safety related parameters	dry-fermented sausages made of beef dry-fermented sausages made of fallow-deer meat	Kononiuk, Karwowska [93] Kononiuk, Karwowska [94]
lactates	antibacterial activity, color development, lower cooking loss	pasteurized canned poultry products	Gajowiecki et al. [99]
*Lactobacillus fermentum* RC4 and *Lactobacillus plantarum* B6 -starters, beetroot and *Monascus –* coloring agents, nisin as antibiotic	antibacterial activity, control lipid oxidation, color development	cured pork meat	Huang et al. [100]
annatto powder	color development control of bacterial growth	cooked sausages	Zarringhalami et al. [101]

## References

[1] WHO (2016). Nitrate and Nitrite in Drinking-Water. Background Document for Development of WHO Guidelines for Drinking-Water Quality.

[2] Ward M.H., Jones R.R., Brender J.D., De Kok T.M., Weyer P.J., Nolan B.T., Villanueva C.M., Van Breda S.G. (2018). Drinking Water Nitrate and Human Health: An Updated Review. Int. J. Environ. Res. Public Health.

[3] Larsson K., Darnerud P.O., Ilbäck N.G., Merino L. (2011). Estimated dietary intake of nitrite and nitrate in Swedish children. Food Addit. Contam. Part A.

[4] Temme E.H.M., Vandevijvere S., Vinkx C., Huybrechts I., Goeyens L., Van Oyen H. (2011). Average daily nitrate and nitrite intake in the Belgian population older than 15 years. Food Addit. Contam. Part A.

[5] Tamme T., Reinik M., Roasto M., Juhkam K., Tenno T., Kiis A. (2006). Nitrates and nitrites in vegetables and vegetable-based products and their intakes by the Estonian population. Food Addit. Contam..

[6] Ding Z., Johanningsmeier S.D., Price R., Reynolds R., Truong V.-D., Payton S.C., Breidt F. (2018). Evolution of nitrate and nitrite content in pickled fruit and vegetable products. Food Control.

[7] Griesenbeck J.S., Steck M.D., Huber J.C., Sharkey J.R., Rene A.A., Brender J.D. (2009). Development of estimates of dietary nitrates, nitrites, and nitrosamines for use with the Short Willet Food Frequency Questionnaire. Nutr. J..

[8] EFSA (2008). Nitrate in vegetables. Scientific opinion of the panel on contaminants in the food chain. EFSA J..

[9] Hord N.G., Tang Y., Bryan N.S. (2009). Food sources of nitrates and nitrites: The physiologic context for potential health benefits. Am. J. Clin. Nutr..

[10] Lucarini M., D’Evoli L., Tufi S., Gabrielli P., Paoletti S., Di Ferdinando S., Lombardi-Boccia G. (2012). Influence of growing system on nitrate accumulation in two varieties of lettuce and red radicchio of Treviso. J. Sci. Food Agric..

[11] Prasad S., Chetty A.A. (2011). Flow injection assessment of nitrate contents in fresh and cooked fruits and vegetables grown in Fiji. J. Food Sci..

[12] World Health Organization (2007). Nitrate and Nitrite in Drinking Water Development of WHO Guidelines for Drinking Water Quality.

[13] Nuñez de González M.T., Osburn W.N., Hardin M.D., Longnecker M., Garg H.K., Bryan N.S., Keeton J.T. (2015). A Survey of nitrate and nitrite concentrations in conventional and organic-labeled raw vegetables at retail. J. Food Sci..

[14] Roila R., Branciari R., Staccini B., Ranucci D., Miraglia D., Altissimo M.S., Mercuri M.L., Haouet N.M. (2018). Contribution of vegetables and cured meat to dietary nitrate and nitrite intake in Italian population: Safe level for cured meat and controversial role of vegetables. Italian J. Food Saf..

[15] Sindelar J.J., Milkowski A.L. (2012). Human safety controversies surrounding nitrate and nitrite in the diet. Nitric Oxide.

[16] Merino L., Darnerud P.O., Toldrá F., Ilbäck N.-G. (2016). Time-dependent depletion of nitrite in pork/beef and chicken meat products and its effect on nitrite intake estimation. Food Additiv. Contam..

[17] Qin L., Liu X., Sun Q., Fan Z., Xia D., Ding G., Qi S. (2012). Sialin (SLC17A5) functions as a nitrate transporter in the plasma membrane. Proc. Natl. Acad. Sci. USA.

[18] Qu X.M., Wu Z.F., Pang B.X., Jin L.Y., Qin L.Z., Wang S.L. (2016). From nitrate to nitric oxide: The role of salivary glands and oral bacteria. J. Den. Res..

[19] Lundberg J.O., Weitzberg E., Gladwin M.T. (2008). The nitrate–nitrite–nitric oxide pathway in physiology and therapeutics. Nat. Rev. Drug Discov..

[20] Leaf C.D., Wishnok J.S., Tannenbaum S.R. (1989). L-arginine is a precursor for nitrate biosynthesis in humans. Bioch. Bioph. Res. Commun..

[21] Van Faassen E.E., Bahrami S., Feelisch M., Hogg N., Kelm M., Kim-Shapiro D.B., Nohl H. (2009). Nitrite as regulator of hypoxic signaling in mammalian physiology. Med. Res. Rev..

[22] Bryan N.S., Ivy J.L. (2015). Inorganic nitrite and nitrate: Evidence to support consideration as dietary nutrients. Nutr. Res..

[23] Doel J.J., Benjamin N., Hector M.P., Rogers M., Allaker R.P. (2005). Evaluation of bacterial nitrate reduction in the human oral cavity. Eur. J. Oral Sci..

[24] Hyde E.R., Andrade F., Vaksman Z., Parthasarathy K., Jiang H., Parthasarathy D.K., Bryan N.S. (2014). Metagenomic analysis of nitrate-reducing bacteria in the oral cavity: Implications for nitric oxide homeostasis. PLoS ONE.

[25] Lundberg J.O., Govoni M. (2004). Inorganic nitrate is a possible source for systemic generation of nitric oxide. Free Rad. Biol. Med..

[26] Tripatara P., Patel N.S., Webb A., Rathod K., Lecomte F.M., Mazzon E., Thiemermann C. (2007). Nitrite-derived nitric oxide protects the rat kidney against ischemia/reperfusion injury in vivo: Role for xanthine oxidoreductase. J. Am. Soc. Nephrol..

[27] Rassaf T., Flögel U., Drexhage C., Hendgen-Cotta U., Kelm M., Schrader J. (2007). Nitrite reductase function of deoxymyoglobin: Oxygen sensor and regulator of cardiac energetics and function. Circ. Res..

[28] Pereira C., Ferreira N.R., Rocha B.S., Barbosa R.M., Laranjinha J. (2013). The redox interplay between nitrite and nitric oxide: From the gut to the brain. Redox Biol..

[29] Ignarro L.J., Byrns R.E., Sumi D., de Nigris F., Napoli C. (2006). Pomegranate juice protects nitric oxide against oxidative destruction and enhances the biological actions of nitric oxide. Nitric Oxide.

[30] Ma L., Hu L., Feng X., Wang S. (2018). Nitrate and nitrite in health and disease. Aging Dis..

[31] Ohshima H., Bartsch H. (1981). Quantitative estimation of endogenous nitrosation in humans by monitoring N-nitrosoproline excreted in the urine. Canc. Res..

[32] Shephard S.E., Schlatter C.H., Lutz W.K. (1987). Assessment of the risk of formation of carcinogenic N-nitroso compounds from dietary precursors in the stomach. Food Chem. Toxic..

[33] Tricker A.R. (1997). N-nitroso compounds and man: Sources of exposure, endogenous formation and occurrence in body fluids. Eur. J. Cancer Prev. Off. J. Eur. Canc. Prev. Org..

[34] De La Pomélie D., Santé-Lhoutellier V., Gatellier P. (2017). Mechanisms and kinetics of tryptophan N-nitrosation in a gastro-intestinal model. Food Chem..

[35] Bartsch H., Pignatelli B., Calmels S., Ohshima H. (1993). Inhibition of nitrosation. Antimutagenesis and Anticarcinogenesis Mechanisms III.

[36] Lunn J.C., Kuhnle G., Mai V., Frankenfeld C., Shuker D.E.G., Glen R.C., Bingham S.A. (2007). The effect of haem in red and processed meat on the endogenous formation of N-nitroso compounds in the upper gastrointestinal tract. Carcinogenesis.

[37] D’Ischia M., Napolitano A., Manini P., Panzella L. (2011). Secondary targets of nitrite-derived reactive nitrogen species: Nitrosation/nitration pathways, antioxidant defense mechanisms and toxicological implications. Chem. Res. Toxicol..

[38] Dalle-Donne I., Rossi R., Colombo R., Giustarini D., Milzani A. (2006). Biomarkers of oxidative damage in human disease. Clin. Chem..

[39] Calcerrada P., Peluffo G., Radi R. (2011). Nitric oxide-derived oxidants with a focus on peroxynitrite: Molecular targets, cellular responses and therapeutic implications. Curr. Pharm. Des..

[40] Li X., Tao R.R., Hong L.J., Cheng J., Jiang Q., Lu Y.M., Hu Y.Z. (2015). Visualizing peroxynitrite fluxes in endothelial cells reveals the dynamic progression of brain vascular injury. J. Am. Chem. Soc..

[41] Alhasawi A., Legendre F., Jagadeesan S., Appanna V., Appanna V. (2019). Chapter 10-Biochemical strategies to counter nitrosative stress: Nanofactories for value-added products. Microbial Diversity in the Genomic Era.

[42] Pacher P., Beckman J.S., Liaudet L. (2007). Nitric oxide and peroxynitrite in health and disease. Physiol. Rev..

[43] Trostchansky A., Rubbo H. (2008). Nitrated fatty acids: Mechanisms of formation, chemical characterization, and biological properties. Free Rad. Biol. Med..

[44] White P.J., Charbonneau A., Cooney G.J., Marette A. (2010). Nitrosative modifications of protein and lipid signaling molecules by reactive nitrogen species. Am. J. Physiol..

[45] Kurutas E.B. (2016). The importance of antioxidants which play the role in cellular response of against oxidative/nitrosative stress: Current state. Nutr. J..

[46] Yang T., Zhang X.M., Tarnawski L., Peleli M., Zhuge Z., Terrando N., Lundberg J.O. (2017). Dietary nitrate attenuates renal ischemia-reperfusion injuries by modulation of immune responses and reduction of oxidative stress. Redox Biol..

[47] Kim H.J., Lee S.S., Choi B.Y., Kim M.K. (2007). Nitrate intake relative to antioxidant vitamin intake affects gastric cancer risk: A case-control study in Korea. Nutr. Canc..

[48] Ward M.H., Heineman E.F., Markin R.S., Weisenburger D.D. (2008). Adenocarcinoma of the stomach and esophagus and drinking water and dietary sources of nitrate and nitrite. Int. J. Occup. Environ. Health.

[49] Keszei A.P., Goldbohm R.A., Schouten L.J., Jakszyn P., van den Brandt P.A. (2012). Dietary N-nitroso compounds, endogenous nitrosation, and the risk of esophageal and gastric cancer subtypes in the Netherlands Cohort Study. Am. J. Clin. Nutr..

[50] Weyer P.J., Cerhan J.R., Kross B.C., Hallberg G.R., Kantamneni J., Breuer G., Lynch C.F. (2001). Municipal drinking water nitrate level and cancer risk in older women: The Iowa Women’s Health Study. Epidemiology.

[51] DellaValle C.T., Xiao Q., Yang G., Shu X.O., Aschebrook-Kilfoy B., Zheng W., Gao Y.T. (2014). Dietary nitrate and nitrite intake and risk of colorectal cancer in the Shanghai Women’s Health Study. Int. J. Cancer.

[52] Grieb S.M.D., Theis R.P., Burr D., Benardot D., Siddiqui T., Asal N.R. (2009). Food groups and renal cell carcinoma: Results from a case-control study. J. Am. Diet. Assoc..

[53] Dubrow R., Darefsky A.S., Park Y., Mayne S.T., Moore S.C., Kilfoy B., Ward M.H. (2010). Dietary components related to N-nitroso compound formation: A prospective study of adult glioma. Cancer Epidemiol. Prev. Biomark..

[54] De Roos A.J., Ray R.M., Gao D.L., Wernli K.J., Fitzgibbons E.D., Ziding F., Checkoway H. (2005). Colorectal cancer incidence among female textile workers in Shanghai, China: A case-cohort analysis of occupational exposures. Cancer Causes Control.

[55] Cross A.J., Ferrucci L.M., Risch A., Graubard B.I., Ward M.H., Park Y., Sinha R. (2010). A large prospective study of meat consumption and colorectal cancer risk: An investigation of potential mechanisms underlying this association. Cancer Res..

[56] Bahadoran Z., Mirmiran P., Ghasemi A., Kabir A., Azizi F., Hadaegh F. (2015). Is dietary nitrate/nitrite exposure a risk factor for development of thyroid abnormality? A systematic review and meta-analysis. Nitric Oxide.

[57] Song P., Wu L., Guan W. (2015). Dietary nitrates, nitrites, and nitrosamines intake and the risk of gastric cancer: A meta-analysis. Nutrients.

[58] Hu J., Mao Y., White K. (2003). Diet and vitamin or mineral supplements and risk of renal cell carcinoma in Canada. Cancer Causes Control.

[59] Aschebrook-Kilfoy B., Shu X.O., Gao Y.T., Ji B.T., Yang G., Li H.L., Ward M.H. (2013). Thyroid cancer risk and dietary nitrate and nitrite intake in the Shanghai women’s health study. Int. J. Cancer.

[60] Van Hecke T., Vossen E., Hemeryck L.Y., Bussche J.V., Vanhaecke L., De Smet S. (2015). Increased oxidative and nitrosative reactions during digestion could contribute to the association between well-done red meat consumption and colorectal cancer. Food Chem..

[61] Bastide N.M., Chenni F., Audebert M., Santarelli R.L., Taché S., Naud N., Kuhnle G.G. (2015). A central role for heme iron in colon carcinogenesis associated with red meat intake. Cancer Res..

[62] Zheng J., Stuff J., Tang H., Hassan M.M., Daniel C.R., Li D. (2018). Dietary N-nitroso compounds and risk of pancreatic cancer: Results from a large case–control study. Carcinogenesis.

[63] Chan T.Y. (2011). Vegetable-borne nitrate and nitrite and the risk of methaemoglobinaemia. Toxicol. Lett..

[64] Weitzberg E., Lundberg J.O. (2013). Novel aspects of dietary nitrate and human health. Ann. Rev. Nutr..

[65] Kapil V., Khambata R.S., Robertson A., Caulfield M.J., Ahluwalia A. (2015). Dietary nitrate provides sustained blood pressure lowering in hypertensive patients: A randomized, phase 2, double-blind, placebo-controlled study. Hypertension.

[66] Berry M.J., Justus N.W., Hauser J.I., Case A.H., Helms C.C., Basu S., Miller G.D. (2015). Dietary nitrate supplementation improves exercise performance and decreases blood pressure in COPD patients. Nitric Oxide.

[67] Ashworth A., Mitchell K., Blackwell J.R., Vanhatalo A., Jones A.M. (2015). High-nitrate vegetable diet increases plasma nitrate and nitrite concentrations and reduces blood pressure in healthy women. Public Health Nutr..

[68] Montenegro M.F., Amaral J.H., Pinheiro L.C., Sakamoto E.K., Ferreira G.C., Reis R.I., Tanus-Santos J.E. (2011). Sodium nitrite downregulates vascular NADPH oxidase and exerts antihypertensive effects in hypertension. Free Rad. Biol. Med..

[69] Amaral J.H., Ferreira G.C., Pinheiro L.C., Montenegro M.F., Tanus-Santos J.E. (2015). Consistent antioxidant and antihypertensive effects of oral sodium nitrite in DOCA-salt hypertension. Redox Biol..

[70] Ling W.C., Mustafa M.R., Vanhoutte P.M., Murugan D.D. (2018). Chronic administration of sodium nitrite prevents hypertension and protects arterial endothelial function by reducing oxidative stress in angiotensin II-infused mice. Vasc. Pharmacol..

[71] Siervo M., Lara J., Ogbonmwan I., Mathers J.C. (2013). Inorganic nitrate and beetroot juice supplementation reduces blood pressure in adults: A systematic review and meta-analysis. J. Nutr..

[72] Ghasemi A., Gheibi S. (2017). Effect of oral nitrate administration on glucose metabolism and inflammation in obese type 2 diabetic rats. 19th European Congress of Endocrinology.

[73] Khalifi S., Rahimipour A., Jeddi S., Ghanbari M., Kazerouni F., Ghasemi A. (2015). Dietary nitrate improves glucose tolerance and lipid profile in an animal model of hyperglycemia. Nitric Oxide.

[74] Gilchrist M., Winyard P.G., Fulford J., Anning C., Shore A.C., Benjamin N. (2014). Dietary nitrate supplementation improves reaction time in type 2 diabetes: Development and application of a novel nitrate-depleted beetroot juice placebo. Nitric Oxide.

[75] Cermak N.M., Hansen D., Kouw I.W., van Dijk J.W., Blackwell J.R., Jones A.M., van Loon L.J. (2015). A single dose of sodium nitrate does not improve oral glucose tolerance in patients with type 2 diabetes mellitus. Nutr. Res..

[76] Zand J., Lanza F., Garg H.K., Bryan N.S. (2011). All-natural nitrite and nitrate containing dietary supplement promotes nitric oxide production and reduces triglycerides in humans. Nutr. Res..

[77] Rammos C., Hendgen-Cotta U.B., Sobierajski J., Bernard A., Kelm M., Rassaf T. (2014). Dietary nitrate reverses vascular dysfunction in older adults with moderately increased cardiovascular risk. J. Am. Coll. Cardiol..

[78] Bondonno C.P., Blekkenhorst L.C., Prince R.L., Ivey K.L., Lewis J.R., Devine A., Hodgson J.M. (2017). Association of vegetable nitrate intake with carotid atherosclerosis and ischemic cerebrovascular disease in older women. Stroke.

[79] Burnley-Hall N., Abdul F., Androshchuk V., Morris K., Ossei-Gerning N., Anderson R., James P.E. (2018). Dietary nitrate supplementation reduces circulating platelet-derived extracellular vesicles in coronary artery disease patients on clopidogrel therapy: A randomised, double-blind, placebo-controlled study. Thromb. Haemost..

[80] Lefer D.J., Bryan N.S., Organ C.L. (2017). Nitrite and Nitrate in Ischemia–Reperfusion Injury. Nitrite and Nitrate in Human Health and Disease.

[81] Coggan A.R., Leibowitz J.L., Spearie C.A., Kadkhodayan A., Thomas D.P., Ramamurthy S., Peterson L.R. (2015). Acute dietary nitrate intake improves muscle contractile function in patients with heart failure: A double-blind, placebo-controlled, randomized trial. Circ. Heart Fail..

[82] Coggan A.R., Leibowitz J.L., Kadkhodayan A., Thomas D.P., Ramamurthy S., Spearie C.A., Peterson L.R. (2015). Effect of acute dietary nitrate intake on maximal knee extensor speed and power in healthy men and women. Nitric Oxide.

[83] Xie L., Mo M., Jia H.X., Liang F., Yuan J., Zhu J. (2016). Association between dietary nitrate and nitrite intake and site-specific cancer risk: Evidence from observational studies. Oncotarget.

[84] Cantwell M., Elliott C. (2017). Nitrates, Nitrites and Nitrosamines from Processed Meat Intake and Colorectal Cancer Risk. J. Clin. Nutr. Diet..

[85] Honikel K.O., Devine C. (2014). Chemical analysis of specific components curing agents. Encyclopedia of Meat Sciences.

[86] Menard C., Heraud F., Volatier J.L., Leblanc J.C. (2008). Assessment of dietary exposure of nitrate and nitrite in France. Food Addit. Contam..

[87] Herrmann S.S., Duedahl-Olesen L., Granby K. (2015). Occurrence of volatile and nonvolatile N-nitrosamines in processed meat products and the role of heat treatment. Food Control.

[88] Bedale W., Sindelar J.J., Milkowski A.L. (2016). Dietary nitrate and nitrite: Benefits, risks, and evolving perceptions. Meat Sci..

[89] Alahakoon A.U., Jayasena D.D., Ramachandra S., Jo C. (2015). Alternatives to nitrite in processed meat: Up to date. Trends Food Sci. Technol..

[90] Jira W. (2004). Chemical reaction of curing and smoking-Part 1: Curing. Fleischwirtstchaft.

[91] Hammes P.W. (2012). Metabolism of nitrate in fermented meats: The characteristic feature of a specific group of fermented foods. Food Microbiol..

[92] Sindelar J.J., Cordray J.C., Sebranek J.G., Love J.A., Ahn D.U. (2007). Effects of varying levels of vegetable juice powder and incubation time on color, residual nitrate and nitrite, pigment, pH, and trained sensory attributes of ready-to-eat uncured ham. J. Food Sci..

[93] Kononiuk A., Karwowska M. (2019). Physicochemical, proteolytic and sensory changes during long-term storage of dry-fermented sausages made from fallow deer meat in comparison to beef. Food Technol. Sci. Qual..

[94] Kononiuk A., Karwowska M. (2020). Comparison of selected parameters related to food safety of fallow deer and beef uncured fermented sausages with freeze-dried acid whey addition. Meat Sci..

[95] Sucu C., Turp G.Y. (2018). The investigation of the use of beetroot powder in Turkish fermented beef sausage (sucuk) as nitrite alternative. Meat Sci..

[96] Bianchin M., Pereira D., Dos Reis A.S., De Florio Almeida J., Dangui L., Da Silva C.D.M., Carpes S.T. (2017). Research Article Rosemary Essential Oil and Lyophilized Extract as Natural Antioxidant Source to Prevent Lipid Oxidation in Pork Sausage. Adv. J. Food Sci. Technol..

[97] Karwowska M., Dolatowski Z.J. (2017). Effect of acid whey and freeze-dried cranberries on lipid oxidation and fatty acid composition of nitrite-/nitrate-free fermented sausage made from deer meat. Asian-Austral. J. Anim. Sci..

[98] Kim T.K., Hwang K.E., Lee M.A., Paik H.D., Kim Y.B., Choi Y.S. (2019). Quality characteristics of pork loin cured with green nitrite source and some organic acids. Meat Sci..

[99] Gajowiecki L., Kotowicz M., Lachowicz K., Dabrowski W., Koronkiewicz A., Zochowska-Kujawska J., Zych A. (2006). Zastosowanie mleczanow do produkcji wyrobow drobiowych niezawierajacych dodatku azotanu [III] sodu. Food Technol. Sci. Qual..

[100] Huang L., Zeng X., Sun Z., Wu A., He J., Dang Y., Pan D. (2019). Production of a safe cured meat with low residual nitrite using nitrite substitutes. Meat Sci..

[101] Zarringhalami S., Sahari M.A., Hamidi-Esfehani Z. (2009). Partial replacement of nitrite by annatto as a colour additive in sausage. Meat Sci..

